# A Song for the Mind: A Literature Review on Singing and Cognitive Health in Aging Populations

**DOI:** 10.3390/brainsci15030227

**Published:** 2025-02-21

**Authors:** Panagiota Tragantzopoulou, Vaitsa Giannouli

**Affiliations:** 1 School of Social Sciences, University of Westminster, London W1B 2HW, UK; g.tragantzopoulou@westminster.ac.uk; 2 School of Psychology, Aristotle University of Thessaloniki, 54124 Thessaloniki, Greece

**Keywords:** singing, choir singing, aging, cognitive decline, dementia, verbal fluency

## Abstract

**Background/Objectives**: As the global population ages, the need for effective nonpharmacological interventions to support cognitive health has become increasingly urgent. Singing has been identified as a promising strategy to enhance cognitive function and emotional well-being in older adults. While substantial research has focused on the neurocognitive benefits of musical training, the specific effects of singing on neuroplasticity and cognition in aging populations remain underexplored. **Methods**: This review synthesizes findings from PubMed, PsycINFO, and Google Scholar to examine the impact of singing on cognitive health, particularly in mitigating cognitive decline and promoting mental well-being. **Results**: Key benefits of singing include improvements in verbal fluency, executive function, and episodic memory. Structural changes such as increased white matter integrity and enhanced auditory–motor integration highlight the potential of singing to stimulate neuroplasticity. Among individuals with dementia, singing fosters episodic memory, mood enhancement, and social connection, while healthy older adults demonstrate improved verbal flexibility and cognitive resilience. However, methodological limitations, such as small sample sizes and cross-sectional designs, preclude definitive conclusions about long-term benefits. **Conclusions**: Future research should explore the specific neural mechanisms underlying these effects, with an emphasis on longitudinal studies and diverse populations. Tailored, inclusive singing programs could address individual cognitive and physical abilities while fostering sustained engagement and social connection. As a low-cost, scalable intervention, singing holds promise for addressing cognitive and emotional challenges associated with aging, offering an accessible avenue to support healthy aging and enhance quality of life across diverse populations.

## 1. Introduction

Cognitive decline is a natural part of aging, significantly impacting older adults and posing challenges for healthy, functional aging [1]. As the elderly population increases globally, it is vital to explore effective interventions to maintain cognitive health and mitigate age-related cognitive decline. Research suggests that individuals who engage in music-making, including singing, may experience more favorable cognitive outcomes as they age. For instance, studies show that older adults who sing or play a musical instrument exhibit improved cognitive abilities, such as enhanced processing speed, better verbal fluency, and improved learning capabilities [2]. Notably, both professional and amateur musicians have shown brain characteristics that appear younger than their actual age, suggesting that music may be associated with a slower rate of aging in the brain [3]. This highlights the potential of singing as a valuable and enjoyable intervention for counteracting cognitive decline.

While aging is often accompanied by brain senescence and subsequent cognitive decline [1], the adult brain retains its capacity for learning and adaptation. Experiences can influence both brain structure and function, a process referred to as experience-dependent brain plasticity [4]. However, the extent to which this plasticity can offset age-related decline remains underexplored, particularly across different populations. Research has shown that the corpus callosum, which links the left and right auditory processing regions, is larger in adult male musicians compared to non-musicians [5], suggesting enhanced interhemispheric connectivity and improved auditory information transfer [6]. Similarly, radial diffusivity within the transcallosal connectivity between the left and right planum temporale is higher in male musicians [7]. Furthermore, the microstructural integrity of the posterior section of the corpus callosum has been associated with attentional timing differences during verbal dichotic listening in young adults [8]. Additionally, amateur singing has been associated with structural differences in the arcuate fasciculus, a white matter tract that connects the temporal and inferior parietal cortices, areas involved in speech and language processing [9,10]. Notably, greater fractional anisotropy in the auditory callosal pathway (specifically, the genu of the corpus callosum) has been tied to superior auditory performance in young adults [11]. While these findings collectively support the potential of musical activities to drive structural and functional changes in the brain, they also highlight critical gaps in our understanding of how these changes translate into cognitive and behavioral outcomes.

Overall, musical activities appear to be associated with functional and structural connectivity differences in the brain. Given the well-documented age-related decline in brain structures, including gray matter [12,13] and white matter integrity [9,12], these structural changes may influence the efficiency and resilience of neural pathways. Musical activities, such as singing and playing instruments, are inherently complex, engaging perceptual and motor systems alongside affective, cognitive, and motivational processes [14]. Performing music demands precise timing, refined motor control, auditory perception, and the integration of auditory and motor functions [15]. Due to this complexity, musical training may extend its influence beyond the auditory system [16], potentially benefiting speech abilities [17], fine motor skills [18], and inhibitory control [19]. These findings align with the mental exercise hypothesis, which posits that mentally stimulating activities can support cognitive health [20,21].

### Background and Rationale

Although instrumental music training’s neurocognitive effects have been widely explored, much less is understood about how singing influences neuroplasticity and cognitive functions, particularly as we age. Singing engages a wide range of brain functions, combining auditory, vocal-motor, linguistic, cognitive, and emotional processes into a dynamic and complex activity [22]. Neuroimaging research indicates that singing involves constant coordination between two major cortical pathways: the dorsal pathway, which connects parietal and frontal regions for vocal production, and the ventral pathway, linking temporal and frontal areas for auditory perception [23]. These pathways form a feedback loop that supports precise vocal motor control by integrating auditory and somatosensory input [23]. Beyond these central systems, additional brain regions—such as the prefrontal cortex, limbic structures, and cerebellum—contribute to aspects like attention, working memory, rhythm, and emotional expression during singing [24]. Given its complexity, singing presents a unique opportunity to investigate its cognitive and emotional benefits in older adults.

Typically, studies tend to compare musicians and non-musicians, defining musicians as individuals who play a musical instrument [25]. Given the potential cognitive and social benefits associated with playing a musical instrument, it raises the question of whether similar advantages might be observed through singing training. Supporting this idea, research by Bialystok and De Pape [26] demonstrated enhanced cognitive performance in young adult instrumentalists and trained vocalists compared to individuals without musical training. Similarly, Mansens et al. [2] in a cross-sectional study involving over 1000 participants aged 64 and older as part of the Longitudinal Aging Study Amsterdam, found that engaging in music-making was positively associated with better cognitive task performance. Notably, while overall cognitive outcomes were similar for instrumentalists and singers, instrumentalists showed significantly higher processing speeds. However, the study did not carefully examine specific aspects of engagement, such as private lessons or group versus solo participation. This leaves open the possibility that individual vocal lessons and structured vocal practice could yield cognitive benefits similar to those observed in private instrumental lessons. The human voice, as a readily accessible “instrument” that does not require ownership of a physical object, may be particularly practical for older adults.

## 2. Methods

Building on this foundation, the primary aim of this review is to explore the potential of singing as a nonpharmacological intervention for enhancing cognitive function and well-being in older adults. While existing studies suggest that musical training, including both instrumental performance and singing, has positive cognitive effects, the specific mechanisms and unique benefits of singing remain less well-understood. To improve this understanding, a comprehensive literature search was conducted using academic databases such as PubMed, PsycINFO, and Google Scholar, focusing on studies published within the last two decades. We employed a systematic approach to selecting studies. The keywords used in the search were informed by previous reviews and relevant literature in the field. These included terms such as “singing”, “cognitive decline”, “neuroplasticity”, “aging”, “music interventions”, and “cognitive function”. The selection of these keywords was based on their direct relevance to the topics of musical interventions and cognitive health in aging populations. Additionally, variations and synonyms of these terms, such as “vocalization” for singing and “cognitive impairment” for cognitive decline, were included to capture a broader range of relevant studies.

**Figure 1:**
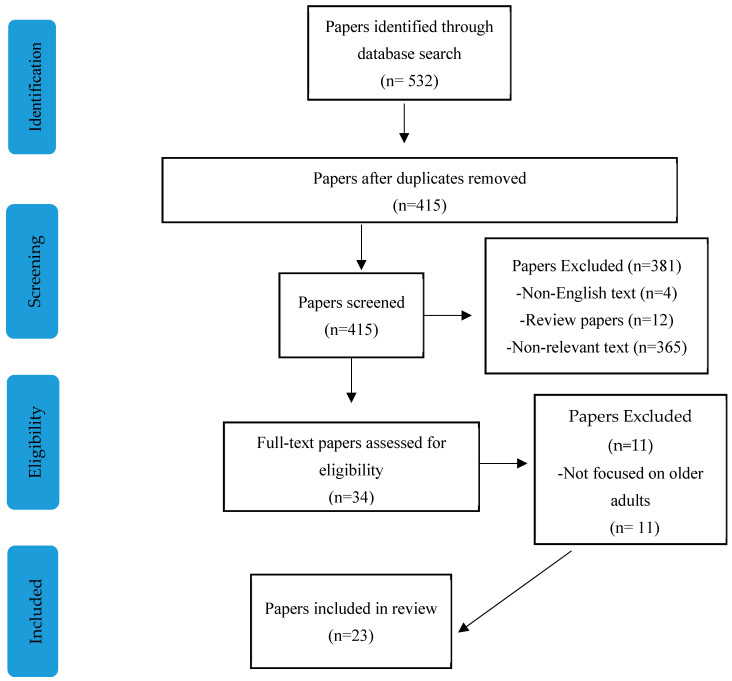
Flowchart.

The inclusion criteria for studies were that they must be peer-reviewed articles published in English within the last 20 years. The studies had to focus on aging adults, specifically those aged 60 and above, and they needed to address the relationship between singing and cognitive health, neuroplasticity, or aging. The studies could be either empirical research or theoretical papers that examined the potential benefits of singing in mitigating age-related cognitive decline. Studies were excluded if they did not focus on cognitive function or well-being, if they did not involve human participants, or if they were published in languages other than English (see Figure 1). Selected studies included both empirical research and theoretical discussions that highlight the potential benefits of singing in mitigating age-related cognitive decline, as well as non-significant findings that help provide a balanced perspective on the effectiveness of singing interventions. By synthesizing these findings, this review aims to bridge existing gaps in the literature, clarify the mechanisms through which singing influences brain and cognitive health, and provide direction for future research. Furthermore, understanding how factors such as group versus individual singing contribute to these effects is essential for advancing the use of singing as an accessible and effective intervention for aging populations.

## 3. Results

### 3.1. Empirical Studies on Singing and Cognitive Health

#### 3.1.1. Singing and Alzheimer’s Disease

Maguire et al. [27] investigated the effects of active singing on cognitive function and life satisfaction among individuals with Alzheimer’s disease (AD) residing in an assisted living facility. The intervention consisted of three vocal music sessions per week, combining familiar and novel songs across various musical genres. Participants, aged 70 to 99 (85% female), included both independent living residents and those in secure-ward settings for dementia. Engagement in the singing groups was monitored throughout the study, categorizing participants as either singers or listeners based on their level of participation. Results indicated that singing was associated with positive impacts, particularly for individuals with dementia. Specifically, singers demonstrated significant improvements in Mini-Mental State Examination (MMSE) and Satisfaction with Life Scale (SWLS) scores compared to listeners, with an interaction effect suggesting that dementia singers outperformed listeners by the study’s conclusion. Additionally, there were improvements in clock-drawing abilities among singers in the dementia group. Overall, findings suggest that an active singing program can lead to meaningful cognitive enhancements and increased life satisfaction, highlighting the potential of music as a nonpharmacological intervention for older adults with cognitive impairments. However, it is crucial to consider potential confounding factors, such as individual differences in baseline cognitive function and motivation levels, which may influence outcomes. Additionally, while results are promising, the absence of a control condition that engages participants in a non-musical activity limits causal inferences.

Building on the evidence that active singing can enhance cognitive function and life satisfaction among individuals with AD, another study explored the effects of structured singing training on cognitive outcomes in patients with AD [28]. A total of ten participants (mean age 78.1 years) engaged in a structured music therapy program that included weekly singing sessions for six months, utilizing karaoke and the YUBA Method, a unique vocal training technique. Neuropsychological assessments were conducted before and after the intervention to evaluate changes in cognitive performance. In addition, functional magnetic resonance imaging (fMRI) was employed to observe brain activity while participants sang familiar songs. A control group of ten AD patients (mean age 77.0 years) underwent neuropsychological evaluations at two intervals over six months without receiving the intervention. The results indicated significant improvements in the music therapy group. Specifically, improved psychomotor processing speed and reduced neuropsychiatric symptoms of dementia after 6 months of weekly singing sessions. However, these results should be interpreted with caution due to the small sample size and the lack of rigorous control over confounding variables such as educational background and baseline cognitive function. While the authors noted that many music therapy studies feature small participant numbers, larger trials are necessary to validate their findings further. Moreover, while fMRI findings offer insights into potential neural mechanisms, they do not directly establish causal relationships between singing and cognitive improvements. Finally, the study focused exclusively on mild to moderate AD patients, highlighting the necessity for additional research to explore the effectiveness of singing training in more severe cases or other forms of dementia, such as vascular dementia.

Another randomized controlled trial (RCT) expanded this scope by examining the effects of choral singing (CSI) compared to a health education program (HEP) on cognitive health in older adults at elevated risk of dementia [22]. Participants attended weekly one-hour sessions for two years, with cognitive performance tracked through a composite cognitive test score (CCTS) and additional biomarkers such as brain imaging, oxidative stress, and immune system health. The results indicated a slight improvement in cognitive scores for the CSI group, while scores declined for the HEP group. This suggests potential cognitive benefits of choral singing. However, after adjusting for initial differences in cognitive scores, the between-group changes were no longer statistically significant. Biomarkers related to brain aging, oxidative damage, and immune health did not show significant differences between the groups, although some brain imaging markers indicated possible trends worth investigating further. The study concludes that choral singing may be as effective as health education in supporting cognitive health in older adults and could serve as a viable intervention for promoting healthy aging. Nonetheless, the study’s design does not allow for a clear differentiation between the specific effects of choral singing and the general effects of social engagement. Future studies should include an additional control group engaged in a different social activity to better isolate the cognitive effects of singing.

Lyu et al. [29] observed significant improvements in semantic verbal fluency immediately after both singing and lyric-reading interventions compared to controls. However, only the singing group maintained these improvements at a 3 month follow-up. A subgroup analysis revealed that participants with mild-stage Alzheimer’s experienced significant gains in immediate and delayed recall at the end of the singing intervention, though these benefits were not sustained after 3 months, suggesting the need for ongoing intervention to preserve cognitive improvements. Similarly, Pongan et al. [30] reported that verbal memory remained stable in participants engaged in a singing intervention, whereas those in a painting control group experienced decline. Both groups showed significant improvements in short-term memory (Digit Span) and processing speed/inhibition (Stroop test), with the singing group showing a trend toward greater improvement in the latter, though this was not statistically significant. The authors noted that assessments were conducted several days to a week after the final intervention, implying that the observed benefits may be more enduring and not merely a result of immediate stimulation. However, given that both groups demonstrated cognitive improvements, it remains unclear whether singing has unique benefits beyond those associated with engaging in any structured group activity.

Several studies have investigated the effects of singing interventions on cognitive function using standardized screening tools, with mixed results. Cooke et al. [31], Camic et al. [32], and Chen et al. [33] all employed the Mini Mental State Exam (MMSE) but did not report any significant overall changes. Camic et al. [32] noted variability in MMSE scores among participants, with some showing improvement while others experienced declines, reflecting the individualized nature of dementia progression. Similarly, Cooke et al. [31] and Maguire [34] observed no significant differences within or between groups, although Maguire [34] reported a non-significant trend toward improved MMSE and Revised MMSE (R-MMSE) scores in participants undergoing an individualized singing intervention (ISI). Additionally, significant gains on other cognitive measures, such as Clock Drawing and Narrative and Complete Sentences tasks, were noted in the ISI group, though these findings require cautious interpretation due to methodological limitations. Chen et al. [33] also found no overall effect of singing on cognition, but reported a significant improvement in the MMSE recall subscale after a group opera singing intervention. A more critical interpretation of these results is necessary, as the reliance on MMSE alone may not adequately capture nuanced cognitive changes. Future research should employ more comprehensive neuropsychological test batteries.

Wang et al. [35] used both the MMSE and the Montreal Cognitive Assessment (MoCA) and observed significant within-group improvements in both the singing and standard care groups, with greater gains in the singing group. Takahashi and Matsushita [36], using the Revised Hasegawa Dementia Scale, found that cognitive function remained stable over a two-year period in participants receiving group singing interventions, whereas a control group experienced a non-significant decline. Notably, participants with moderate-to-high baseline cognitive function showed improvement during the program, though these findings were limited by the small sample size and non-randomized study design. Finally, Davidson and Fedele [37], employing the Hierarchical Dementia Scale in a smaller-scale study, reported no significant changes in cognition following group singing sessions.

#### 3.1.2. Caregiver Singing Intervention for Dementia

In addition to exploring choral singing, another study shifted the focus to caregiver involvement by examining the effects of caregiver singing interventions for individuals with dementia. This intervention aimed to evaluate the effectiveness of a novel music intervention focused on coaching caregivers of persons with dementia (PWDs) to incorporate singing or music listening into their everyday care routines [38]. A total of 89 PWD-caregiver dyads were randomly assigned to one of three groups: a singing coaching group (n = 30), a music listening coaching group (n = 29), or a usual care control group (n = 30). The intervention lasted for 10 weeks and included coaching sessions that primarily involved singing or listening to familiar songs, occasional vocal exercises, and rhythmic movements for the singing group, while the music listening group engaged in reminiscence discussions. The study found that both singing and music listening significantly improved mood, orientation, and remote episodic memory compared to the usual care group. Additionally, singing demonstrated enhancements in short-term and working memory, as well as caregiver well-being, while music listening positively influenced the QOL of caregivers. However, it is crucial to differentiate between cognitive rehabilitation and lifestyle-based interventions, as the long-term effects of caregiver-led singing on cognitive function remain unclear. These results suggest that regular musical activities can yield long-term cognitive, emotional, and social benefits for individuals with mild to moderate dementia and highlight the potential of these interventions in dementia care and rehabilitation. However, the study is not without its limitations. One notable constraint is the lack of focus on specific types of dementia, which may limit the ability to draw specific conclusions about the intervention’s effectiveness for particular dementia populations, such as those with AD. Conversely, this broad focus provides a more representative sample of the wider PWD population, enhancing the generalizability of the findings. Another limitation is the exclusion of participants in the early stages of dementia, where regular musical activities might have the most significant impact. This raises questions about the optimal timing for implementing music interventions to achieve the best long-term outcomes.

### 3.2. Singing and Cognitive Benefits in Healthy Adults

#### 3.2.1. Verbal Fluency and Memory

Research into the cognitive benefits of singing in healthy older adults suggests promising but preliminary findings. In a pilot study involving 49 healthy older participants, Fu et al. [39] observed improvements in verbal fluency and memory following a 12 week group singing program. However, the absence of a well-matched control group significantly limits the interpretability of these results, as it remains unclear whether the observed changes were directly attributable to the singing intervention or other factors. Expanding on this, a cross-sectional study by another research group investigated the potential cognitive, emotional, and social benefits of choir singing in 162 older adults, comparing choir singers with non-singers across several domains, including cognitive functioning, mood, quality of life, and social integration [40]. Participants included individuals with varying levels of choir experience, and a subset of 74 singers completed more detailed neuropsychological assessments. The findings revealed that choir singers performed better in verbal flexibility, a key aspect of executive function, though no significant differences were found in other cognitive domains. Notably, choir singers with high activity levels reported stronger social connections, while those with less choir experience reported better general health. Although these findings suggest cognitive and social benefits, their reliance on self-selection limits causal inference. Future longitudinal studies with robust controls are necessary to better understand the long-term effects of choir singing on cognitive and social well-being in aging populations.

In parallel, a recent study explored the relationship between amateur singing and speech production, focusing on both younger and older adults [41]. The researchers aimed to determine whether amateur singing experience could enhance speech articulation, potentially offering insights into therapeutic models for individuals with speech deficits. The study involved 78 participants, including 38 amateur singers and 40 non-musician controls, spanning ages 20 to 88. Participants completed a series of tasks assessing vocal and oral motor abilities, such as voice production, passage reading, and diadochokinetic (DDK) rates at both natural and rapid speeds. The findings revealed that aging was associated with declines in reading and articulation rates, as well as reduced accuracy in phonologically complex tasks. However, amateur singers outperformed non-musicians in articulatory accuracy, particularly in more demanding scenarios. This suggests that amateur singing could be a viable intervention for speech rehabilitation, particularly in populations with speech impairments.

Additionally, musical activities are increasingly recognized for their role in supporting cognitive well-being in older adults, though the long-term effects of specific musical activities such as singing remain underexplored. Addressing this gap, a longitudinal study examined the effects of choir singing on cognitive, emotional, and social well-being in older adults over a two-year period [42]. The study involved 107 choir singers and 62 matched non-singers, collecting data at baseline, one-year, and two-year intervals. Assessments included self-reported measures of cognitive functioning, depression, social engagement, and quality of life (QOL), as well as neuropsychological testing in a subset of participants. The findings revealed that choir singers demonstrated sustained higher performance in verbal flexibility (as measured by a phonemic fluency task) compared to non-singers, who improved over time but started at a lower baseline. Additionally, while both groups exhibited changes in word knowledge retrieval and environmental quality-of-life perceptions, the trends differed, with singers showing improvements and non-singers experiencing declines. Notably, participants newer to choir singing displayed greater gains in verbal skills, suggesting benefits associated with initiating singing later in life. However, no significant differences were found in other cognitive, emotional, or social measures. These results highlight that choir singing may offer specific cognitive advantages, particularly in verbal flexibility, for older adults. However, the findings regarding broader impacts on verbal skills and quality of life remain inconclusive, warranting further investigation into the mechanisms and variability of these effects. Future research should focus on the mechanisms underlying these benefits and whether structured interventions can amplify these effects.

#### 3.2.2. Executive Function and the Brain

Singing, particularly in amateur contexts such as choir participation, has emerged as a promising activity for supporting cognitive functioning in older adults. Research has demonstrated specific cognitive benefits associated with singing, particularly in domains related to executive functions. Joyal and colleagues [43] investigated how musical activities, including singing, influence key components of executive functions—such as selective attention, inhibitory control, and working memory—across a lifespan. Findings indicated that while age-related declines in executive functions were apparent in all participants, singers consistently outperformed non-musician controls in certain domains. For instance, singers demonstrated superior verbal working memory, which researchers attributed to the unique cognitive demands of learning and memorizing lyrics [43]. This activity may foster a “near transfer” effect, enhancing memory performance in contexts closely related to the act of singing. However, these benefits were not universally observed across all cognitive domains, highlighting the domain-specific nature of the cognitive advantages associated with singing. Further exploration is needed to determine how these cognitive advantages translate to daily functioning and whether they persist in advanced aging.

Importantly, the study also found that the cognitive advantages observed in singers were independent of age, suggesting that singing does not necessarily slow the rate of cognitive decline but instead provides a stable enhancement in specific cognitive functions throughout adulthood [43]. Additionally, the social and multisensory environment of choir singing, which involves coordination with other singers and attentiveness to musical cues, appeared to influence attention processes [43]. Interestingly, this social and cognitive stimulation may also introduce challenges, as singers exhibited increased distractibility compared to instrumentalists and non-musicians. This finding underscores the nuanced relationship between singing and cognitive performance, suggesting both potential benefits and cognitive demands inherent in the activity.

In addition to its cognitive effects, singing has been shown to support structural brain health. Another study explored the relationship between lifetime participation in choir singing and brain structure, particularly white matter (WM) integrity and gray matter (GM) volume [44]. The results indicated that amateur choir singing was strongly associated with enhanced WM microstructure across a range of commissural, associative, and projection pathways. These enhancements were observed across the adult lifespan, with singers demonstrating increased connectivity in regions related to language processing, emotional regulation, and motor function. For older adults specifically, choir singing was associated with selective improvements in the microstructure of the fornix, a key white matter tract linked to memory and limbic system function [44]. These findings suggest that singing may provide a degree of structural support for age-typical brain changes, particularly in tracts critical for memory and emotional health.

Interestingly, despite the observed enhancements in white matter integrity, no significant associations were found between choir singing and gray matter volume. This finding suggests that while singing appears to bolster structural connectivity in the brain, it may not directly counteract the gray matter atrophy commonly observed with aging. Nonetheless, the improvements in white matter pathways underscore the potential of singing to promote neural connectivity, a factor that is closely linked to overall cognitive resilience and reserve. The enhanced connectivity observed in older singers is particularly promising, as it suggests that lifelong participation in musical activities like singing may provide a protective benefit for brain health, even in the later stages of life.

Taken together, these studies highlight the potential of singing, particularly in the form of group-based activities like choir participation, to serve as a cognitively enriching intervention for older adults. The activity combines cognitive, social, and emotional stimulation, making it a multidimensional approach to supporting both brain structure and function. While the cognitive benefits appear to be domain-specific and stable across the lifespan, the structural enhancements in white matter integrity, particularly in key pathways such as the corpus callosum and fornix, suggest that singing holds significant promise for maintaining cognitive and neural health in aging populations. These findings contribute to a growing body of evidence supporting the use of music-based interventions to promote cognitive reserve and mitigate age-related cognitive decline.

#### 3.2.3. Auditory Perception, Information Processing, and Inhibitory Control

Recent studies exploring the effects of singing on cognitive and neural health have provided growing evidence for its potential to mitigate age-related declines in cognitive and auditory processing. One notable area of investigation involves speech perception in noise (SPiN), a critical aspect of auditory cognition that often declines with age. Research utilizing surface-based morphometry and moderated mediation analysis has revealed that amateur choral singing is associated with structural plasticity in auditory and dorsal speech stream regions, which in turn supports SPiN performance [45]. This association, however, is contingent on specific singing practices, including frequent practice at home, participation in group singing for three or more hours per week, singing in multiple languages, and receiving formal singing training. These findings suggest a dose-dependent relationship, wherein greater intensity and diversity of singing practice yield greater benefits. It is important to note that not all singing practices confer the same benefits and the absence of a significant overall difference in SPiN performance between singers and non-singers highlights the importance of individual practice behaviors and intensity in driving the observed cognitive advantages.

Furthermore, the study underscores the role of auditory–motor integration in mediating these benefits. Specific singing profiles, such as those involving formal training and multilingual singing, appear to reinforce speech motor representations, facilitating improved discrimination of speech sounds under challenging listening conditions [45]. Interestingly, the frequency of practice emerged as a more critical determinant of SPiN improvement than the overall duration of singing sessions, aligning with prior research on neuroplasticity. This supports the OPERA hypothesis, which posits that repetitive practice is essential for inducing lasting neural adaptations [17]. Thus, while choral singing shows promise as an intervention for maintaining auditory cognitive functions in aging, its efficacy appears to depend on the intensity, diversity, and frequency of practice, highlighting the need for personalized approaches in musical interventions.

In addition to its effects on auditory cognition, singing also influences brain connectivity, offering new insights into the neuroplastic mechanisms underlying cognitive aging. Resting-state functional connectivity (RSFC) analyses have demonstrated differences in brain physiology between singers and non-singers across several networks, including the auditory, speech, language, default mode, and dorsal attention networks [46]. Notably, connectivity within the default mode network (DMN), particularly in the precuneus, was associated with auditory cognitive performance. Lower RSFC in this region was paradoxically linked to better auditory cognition in both singers and non-singers, suggesting that specific patterns of functional connectivity may confer cognitive advantages. These findings provide evidence that singing may influence intrinsic brain connectivity in ways that support auditory and cognitive resilience during aging. While RSFC was not universally lower with age, the observed differences between singers and non-singers highlight the potential of singing to modulate brain networks associated with cognitive function.

Beyond auditory cognition, singing has also been linked to broader cognitive benefits, including faster information processing, better inhibitory control, and enhanced auditory frequency discrimination in both young and older adults [47]. These findings suggest that singing engages cognitive domains critical for efficient decision-making and attentional control, which are often susceptible to decline with aging. The multisensory and motoric demands of singing, coupled with its repetitive and structured nature, may stimulate executive functions and auditory processing pathways, thereby enhancing overall cognitive performance.

Taken together, these studies provide compelling evidence that singing can positively influence cognitive aging through a combination of structural and functional neural adaptations. While the benefits of singing appear to be contingent on specific practice behaviors, its capacity to promote auditory–motor integration, enhance resting-state brain connectivity, and support executive function underscores its potential as a multidimensional cognitive intervention. Further research is needed to elucidate the mechanisms underlying these effects and explore the extent to which personalized singing programs can optimize cognitive and neural health in aging populations. It would also be crucial to address methodological concerns, such as small sample sizes and the control of confounding variables. This would help clarify the specific impacts of singing on cognitive health and ensure robust conclusions.

### 3.3. Choral Singing and Mental Health in Older Adults

Older adults often face increased risks of loneliness and social isolation, which can negatively impact their emotional, cognitive, and physical health [48]. In one of the first RCTs targeting the mental health and quality of life of older adults, 258 participants aged 60 and above were involved in the research conducted in England [49]. The findings suggest that community singing significantly enhances mental health-related QOL for older adults and may serve as a valuable intervention for maintaining mental well-being. Choral singing interventions for non-demented elderly individuals demonstrate psychological benefits, including improved morale, decreased loneliness, enhanced mental health-related QOL, and reduced levels of anxiety and depression compared to control groups. Participants expressed high levels of enjoyment and satisfaction with the singing experience, with many groups continuing to meet after the study’s conclusion. However, the study was conducted in a predominantly white British area, which raises questions about the generalizability of the findings to other demographics. Furthermore, the relatively short duration of the intervention raises concerns about the potential for sustained benefits from longer engagement in singing activities.

This study explored the effects of choir singing on the QOL in older adults by comparing 109 choir singers with 307 matched controls from the general population in Finland [50]. After adjusting for sociodemographic factors, health satisfaction, and engagement in hobbies, the choir singers reported significantly higher physical QOL but no notable differences in psychological QOL compared to controls. Choir singers were also 1.5–1.6 times more likely to report higher overall QOL and satisfaction with health. However, the study’s limitations include its small, region-specific sample and cross-sectional design, which limit generalizability and preclude causal conclusions. Nonetheless, the findings suggest that choir singing may enhance physical well-being in older adults, highlighting the need for further research to understand the long-term and causal impacts.

## 4. Discussion

This paper explored the cognitive and emotional benefits of singing, with a focus on its applications for both healthy adults and individuals with cognitive impairments such as dementia. The review reveals a robust yet nuanced understanding of the potential benefits of singing interventions (see Table 1), as well as the gaps that remain in current research.

The findings suggest that singing, particularly in group settings such as choirs, may be associated with cognitive engagement and social connection, although the directionality and causal nature of these relationships remain uncertain. Studies among healthy older adults demonstrate improvements in verbal flexibility and executive function, with specific enhancements observed in verbal fluency tasks [39,40,41]. Furthermore, the structural and functional benefits of singing on brain health, such as increased white matter integrity and changes in resting-state functional connectivity [44,46], underscore its potential as a tool for cognitive enrichment. However, it is important to emphasize that the studies investigating these neurobiological changes primarily show associations between behavior and biomarkers, and should not be used to claim direct causal relationships between singing and cognitive enrichment, as this has not been conclusively established. Similarly, among individuals with dementia, singing has been shown to enhance episodic memory and mood, contributing to overall well-being [22,27,28]. These findings align with the broader literature suggesting that the multimodal demands of singing—integrating physical, cognitive, and emotional elements—stimulate neuroplasticity and may mitigate age-related cognitive decline [2,5,6].

Despite these promising outcomes, the evidence remains limited by methodological constraints. Several studies rely on small sample sizes, lack control groups, or employ cross-sectional designs, which preclude causal inferences about the long-term benefits of singing. For instance, while improvements in verbal flexibility and social well-being are evident in healthy older adults [39,40,41,42], many studies do not measure other cognitive domains, leaving the broader cognitive impact uncertain. Similarly, while structural benefits in white matter integrity have been observed [44], the absence of significant gray matter changes warrants further investigation into the specific neural mechanisms underlying these benefits. An additional consideration is the variability in outcomes based on participants’ baseline characteristics, including prior musical experience, socioeconomic background, and overall health.

To maximize the benefits of singing as a tool for promoting health and well-being among older adults, several practical implementation strategies should be prioritized. Community-based singing programs should be expanded and tailored to diverse populations, including individuals from low socioeconomic backgrounds or those with limited access to recreational activities. Local community centers, senior living facilities, and healthcare providers can collaborate to establish inclusive choir initiatives that accommodate varying levels of musical experience and physical ability. Offering free or subsidized programs, transportation services, and flexible scheduling can help overcome barriers to participation and foster sustained engagement. These programs should be inclusive, accommodating varying levels of musical experience, and designed to provide sustained engagement, fostering a sense of community and social connection. Additionally, tailored singing interventions should also account for participants’ baseline cognitive and physical abilities. For example, programs designed for individuals with dementia could include simplified songs and memory-focused activities that stimulate recall and emotional connection [51], while those for cognitively healthy older adults might include more complex musical arrangements to challenge executive function and cognitive flexibility. Healthcare providers and activity coordinators in care homes can incorporate singing sessions into daily schedules, ensuring that participants have regular opportunities to engage in this enriching activity. For scalability, integrating singing programs into existing healthcare settings offers significant promise. Memory care units, rehabilitation centers, and outpatient clinics can adopt singing-based activities as part of their therapeutic offerings. Structured group singing sessions led by trained facilitators, such as music therapists or choir directors, could address specific health outcomes, including reducing anxiety, enhancing speech articulation, or improving respiratory function. These sessions could be supplemented with digital tools, such as virtual choir platforms or mobile apps, enabling participants to engage from the comfort of their homes.

Long-term studies are also needed to examine the effects of sustained participation in singing on cognitive trajectories, emotional health, and neural plasticity [52]. Such research could help identify optimal intervention durations and frequencies, providing evidence-based guidelines for program design. Comprehensive assessments should be integrated into research and practice to capture the multifaceted effects of singing. These measures could include neuroimaging, blood biomarkers, cognitive testing, and qualitative interviews, offering insights into the mechanisms underlying observed benefits and allowing for a more nuanced understanding of singing’s impact. Collaboration between neuroscientists, music therapists, public health practitioners, and healthcare providers is vital for designing and implementing targeted singing interventions. Such partnerships can ensure that programs are evidence-based, culturally sensitive, and capable of addressing diverse health challenges [52].

The findings and strategies outlined have significant implications for both individual and public health. Singing programs are inherently low-cost and scalable, making them accessible interventions for promoting active aging and addressing cognitive and social challenges associated with aging populations [49]. Furthermore, integrating singing into healthcare settings—such as memory care units or rehabilitation programs—can complement existing therapies, providing cognitive and emotional support in a structured, engaging format [52]. Finally, emphasizing inclusivity and diversity in program design highlights the potential for singing to address health disparities. By offering a culturally adaptable and universally engaging activity, singing programs can bridge gaps in access to health-promoting resources, creating opportunities for older adults from all backgrounds to experience the cognitive, emotional, and social benefits of this multimodal activity.

## 5. Conclusions

In conclusion, singing represents a promising avenue for fostering cognitive and emotional resilience in aging populations. However, it is important to recognize that the effectiveness of singing interventions may differ between healthy aging and clinical aging populations. While existing research shows the potential benefits of singing for cognitive and emotional well-being in healthy older adults, the impact on clinical populations, such as those with Alzheimer’s disease or other forms of dementia, may require more tailored approaches due to the distinct neurodegenerative patterns and cognitive impairments that characterize these conditions. Future studies should address the methodological limitations currently present in the field. Utilizing advanced neuroimaging techniques such as fMRI, EEG, and connectomics could provide more precise insights into the neural mechanisms underlying the observed benefits of singing. Additionally, longitudinal studies with larger, more diverse populations, including both healthy and clinical aging groups, are essential to establish the long-term effects and generalizability of these findings. New areas of inquiry could explore how variables such as cultural context, musical training, and group dynamics influence the cognitive and emotional outcomes of singing interventions. Interdisciplinary collaborations between neuroscientists, psychologists, music therapists, and gerontologists could further enrich the understanding of how singing can support both healthy aging and clinical populations with cognitive impairments. Finally, integrating technological innovations, such as virtual singing programs or apps, could expand access and participation among older adults, even in remote or underserved areas. By addressing these gaps and exploring new dimensions, future research can harness the full potential of singing to enhance quality of life and support both healthy aging and clinical aging across diverse populations.

## Figures and Tables

**Table 1:** Summary of Singing Benefits.

Domain	Specific Benefit	Example or Mechanism
Verbal Working Memory	Enhanced verbal memory performance	Memorization of lyrics
Executive Function	Improved selective attention, inhibitory control, and verbal fluency	Focused attention during singing tasks, better decision-making
Speech Perception in Noise	Better ability to perceive speech in noisy environments	Stronger effect among formally trained choral singers
White Matter Integrity	Enhanced microstructure of white matter in pathways related to memory and emotional regulation	Neural connections supporting memory and emotion
Auditory–Motor Integration	Better discrimination of speech sounds, linked to cognitive aging benefits	Coordination of auditory and motor systems
Cognitive Flexibility	Sustained improvements in verbal flexibility, aiding in word retrieval, phonemic fluency, and memory functions	Collaborative choir singing enhances adaptability in linguistic tasks
